# The Epidemiology of HIV and Prevention Needs of Men Who Have Sex with Men in Abidjan, Cote d’Ivoire

**DOI:** 10.1371/journal.pone.0125218

**Published:** 2015-04-24

**Authors:** Avi J Hakim, Josephine Aho, Gisele Semde, Mamadou Diarrassouba, Konan Ehoussou, Bea Vuylsteke, Christopher S. Murrill, Marguerite Thiam, Therese Wingate, SHARM Study Group

**Affiliations:** 1 Division of Global HIV/AIDS, U.S. Centers for Disease Control and Prevention, Atlanta, Georgia, United States of America; 2 Institute of Tropical Medicine, Antwerp, Belgium; 3 FHI 360, Abidjan, Côte d’Ivoire; 4 Division of Global HIV/AIDS, U.S. Centers for Disease Control and Prevention, Abidjan, Côte d’Ivoire; 5 Ministry of Health and the Fight Against HIV, Abidjan, Côte d’Ivoire; Emory University Rollins School of Public Health, UNITED STATES

## Abstract

To determine HIV prevalence and associated risk factors among men who have sex with men (MSM) in Abidjan, Côte d’Ivoire. We conducted a cross-sectional RDS survey of MSM in Abidjan from October 2011 to February 2012. Eligibility criteria included age ≥ 18 years and having had oral or anal sex with another man in the last 12 months. Weighted data analysis was conducted with RDSAT and SAS. We enrolled 603 participants, of whom 601 (99.7%) completed the questionnaire and 581 (96.7%) consented to HIV testing. HIV population prevalence was estimated as 18.0% (95% CI: 13.0-23.1); 86.4% (95% CI: 75.1-94.9) of HIV-positive MSM were unaware of their serostatus. In multivariable analysis, adjusting for age, education, and income, HIV infection was associated with unprotected sex at last sex with a woman, more than two male anal sex partners in last 12 months, inconsistent condom use during anal sex with a man, self-perceived risk of HIV, history of forced sex, history of physical abuse due to MSM status, and not receiving last HIV test result prior to study. HIV prevalence among MSM in Abidjan is more than four times as high as that of general population men. MSM engage in high-risk sexual behavior and most HIV-positive MSM are unaware of their serostatus. Greater access to HIV prevention, care, and treatment services targeted to MSM is necessary.

## Introduction

Men who have sex with men (MSM) have long been recognized as being at high risk of HIV [1–3]. Surveys among MSM in the developing world have shown that the risk of HIV infection is up to 19.3 times as high in MSM as in the general population [4]. MSM in Africa are especially vulnerable to HIV due to stigma, laws against homosexuality, and limited access to HIV services [5]. As reflected by the growing number of HIV surveys targeting MSM, sub-Saharan African countries are beginning to acknowledge the impact of HIV among MSM and have provided at least tacit recognition that MSM must be included in the fight against HIV [6]. These surveys have shown, however, the human cost of this delay. Recent surveys in sub-Saharan Africa have reported MSM HIV prevalence ranging from 10.9% in South Africa to 24.5% in Kenya [5, 7–11]. Most of these studies were conducted in Southern and East Africa, where HIV prevalence in the general population is also high.

Côte d’Ivoire is a West African country with an HIV prevalence estimated at 3.7% in the general population, the second highest prevalence in West Africa [12]. While homosexuality is not illegal in Côte d’Ivoire, MSM still face stigmatization and challenges accessing appropriate HIV services. A 2007 survey of 96 male sex workers, the majority of whom were MSM, receiving services at an HIV/STI clinic in Abidjan, Côte d’Ivoire, yielded an HIV prevalence of 50% [13]. We conducted a bio-behavioral survey to determine HIV prevalence and associated risk factors of MSM in Abidjan. This is among the first large-scale MSM surveys in West Africa.

## Methods

### Study Design, Participants, and Setting

This survey had a cross-sectional design and utilized respondent-driven sampling (RDS) to recruit MSM [14–17]. Eligibility criteria included age ≥ 18 years, history of oral or anal sex with another man in the previous 12 months, residence in Abidjan for the previous six months, and ability to communicate in French. The target sample size of 600 was calculated using an estimated HIV prevalence of 20% among MSM, a 95% confidence with a width of 10%, and a design effect of 2.

A formative assessment was conducted in August 2009 to inform survey methods, acceptability of the survey to MSM, and access to services [18]. The assessment revealed that MSM are stigmatized and have few public venues to congregate. Older MSM and non-African MSM are especially hidden and would be difficult to recruit. MSM have rich and diverse social networks spanning across Abidjan, suggesting RDS a useful recruitment method. MSM were willing to recruit peers and participate in the bio-behavioral survey contingent upon MSM involvement in the survey process and mobilization efforts that communicate survey benefits to individuals and the MSM population at large. MSM further reported limited access to HIV and STI services.

Due to the political crisis and violence in Côte d’Ivoire the survey was postponed for one year. Enrollment occurred between October 2011 and February 2012. Data collection ceased for 11 days between Christmas and the New Year without adverse effect on recruitment. Two study sites, one in the north and one in the south side of Abidjan, operated on alternate days from Tuesday to Saturday. Potential seeds were identified during the formative research phase. MSM were asked to refer potential seeds to the investigators and indicate their own interest in being a seed. Potential seeds were screened to determine their support of the survey objectives and the extent of their social networks. Six seeds were recruited to start the survey. They were purposively selected to diversify the following characteristics: age, residence, education, income, occupation, and HIV status. Participants could visit either site and were given three coupons each to recruit MSM peers. Recruitment instructions to seeds and non-seeds included to invite the first three MSM encountered and whom they already knew to visit a study site within two weeks of the recruiter’s first visit. Homophily and recruitment matrixes were monitored to identify recruitment bottlenecks. Five seeds were added later due to poor performance of some referral chains and to increase recruitment among under-represented sub-groups, including older MSM. Survey staff ceased giving participants coupons when the sample size approached the target of 600. To determine personal network size, a cascade of questions was posed asking the number of: MSM known in Abidjan, aged ≥ 18 years, seen in the last 30 days.

### Data Collection

During the first visit potential participants were screened for eligibility and consented before enrolling in the survey. Participants were administered a face-to-face computer-assisted personal interview (QDS v2.6.1, Nova Research, Bethesda, USA) to collect information on their demographics, risk factors for HIV, and access to services. Participants provided a finger-prick blood specimen for rapid HIV testing. At the first visit, HIV results were communicated to participants and STD treatment given to those with symptoms according to the national treatment protocol. All participants were asked to return to the study site in two weeks to collect their secondary incentives for recruiting other MSM, and to participate in a short interview about their recruitment efforts. A unique non-identifying secret code was developed for each participant to verify their identity at the second visit. Participants received 3,000 FCFA (500 FCFA = $1 US) for transport at each visit, 2,000 FCFA for time at the first visit, and 1,000 FCFA for each participant recruited. Urine and rectal swabs were collected for gonorrhea and chlamydia testing and may have served as an additional incentive to participation and for returning for a second visit to receive test results and referral to treatment as appropriate. Participants also received condoms, lubricants, and MSM-specific HIV education material.

### Laboratory methods

Rapid HIV testing was conducted at the study site according to the national algorithm of serial testing using Determine (Abbott Inc., Chicago, USA), Bioline (Standard Diagnostics Inc., Suwon, South Korea), and Statpak (Chembio Diagnostic Systems, Medford, USA). Determine was used as the screening test with Bioline as the confirmatory test. Statpak was used if Determine yielded a reactive result and Bioline a non-reactive result. The result of the Statpak test was considered to be final.

### Analysis

The personal network size used for analysis purposes was the number seen in the last 30 days. RDS Coupon Manager (RDSCM 3.0, CDC, Atlanta, Georgia) was used to track the relationship between recruiter and recruit, and the number of coupons distributed and returned. Interview data were checked for consistency and errors, and cleaned using SPSS 17.0 (SPSS Inc., IBM, Chicago, US). Seeds were included in all analyses. Univariate and bivariate analyses calculated the RDS-I estimator using Respondent Driven Sampling Analysis Tool (RDSAT) 6.0.1 (Cornell University, Ithaca, US, www.respondentdrivensampling.org). We modified our settings to use the dual component network size estimation, a bootstrap with 15,000 samples, a 95% confidence interval, and enhanced data smoothing. Additional bivariate and multivariable analyses were conducted using SAS 9.3 (SAS Institute Inc., Cary, USA) using survey procedures and clustering by seed. HIV infection was the primary outcome of interest and individual HIV sampling weights were used for bivariate and multivariable analyses. All variables, regardless of significance, were and adjusted for age, education, and income in multivariable analysis. Unless otherwise indicated, all data presented are weighted to adjust for RDS study design and represent the larger MSM population in Abidjan beyond study participants.

### Ethical Considerations

Participants provided written consent for survey participation and provision of each biological specimen. The study received ethical approval from the Côte d’Ivoire National Ethics Committees, the US Centers for Disease Control and Prevention, FHI360, and the Institute for Tropical Medicine in Antwerp, Belgium. The survey was also monitored daily by the Survey Coordinator, and spot checks were performed by other investigators.

## Results

### Sampling

Of the 1,608 coupons distributed, 666 were redeemed and 643 were valid. Forty people (16.1%) were deemed ineligible and two seeds did not recruit anyone. Of the 40 ineligible individuals, last sex with a man was more than 12 months prior for 11 and another 11 were found not to be MSM. Of the 603 people enrolled in the study, 601 completed the interview, of which 90.8% returned for the second visit. Blood specimens for HIV testing were provided by 582 participants. All participants reported their personal network size. The maximum number of waves was 12 and equilibrium for HIV was reached at wave 2. Older and younger MSM tended to recruit MSM from within their age group. University-educated MSM had a tendency to recruit similarly educated MSM, while those with only a primary education tended to recruit MSM with an education level different from their own. The estimated design effect for HIV was 2.65 [19].

### Key Characteristics of MSM in Abidjan

Demographic and social characteristics of MSM in Abidjan are presented in Table 1. More than three-quarters of MSM were between ages 18 and 30, and 71.9% earn less than 100 USD a month. More than half (57.6%) self-identify as bisexual. Another 40.2% self-identify as homosexual.

**Table 1:** Demographic and Social Characteristics of MSM in Abidjan, Côte d’Ivoire, 2011–2012 (n = 601).

Variables	Sample Prevalence % (n)	Estimated Population Prevalence % (95% CI)
**Age (median 23, range 18–51)**		
18–24	59.1 (355)	63.9 (57.2–70.5)
25–29	25.8 (155)	23.8 (18.5–29.4)
30–34	9.1 (55)	8.7 (5.1–12.1)
35–39	3.8 (23)	2.8 (1.2–5.5)
40+	2.2 (13)	0.8 (0.1-.7)
**Monthly income in FCFA (500 FCFA = 1 USD)**		
< 25,000	39.1 (234)	47.4 (41.1–53.8)
25,000–49,999	25.9 (155)	24.5 (19.5–29.7)
50,000–149,999	24.1 (144)	20.6 (16.3–25.3)
150,000–299,999	7.2 (43)	4.5 (2.3–6.9)
≥ 300,000	3.7 (22)	2.8 (1.2–4.7)
**Highest education level started**		
Never been to school	5.8 (35)	6.3 (3.5–8.8)
Primary	7.0 (42)	9.3 (5.8–13.1)
Secondary	55.9 (336)	59.6 (54.3–66.7)
Post-secondary	31.3 (188)	24.8 (19.1–29.7)
**Work status**		
Unemployed	14.5 (87)	17.5 (12.5–22.2)
Student	40.4 (243)	40.6 (34.7–46.6)
Shopkeeper, retailer, hotel worker	18.6 (112)	14.5 (10.8–19.2)
Laborer, driver, artist	15.0 (90)	17.2 (12.4–22.7)
Clerical, professional	5.1 (31)	4.6 (2.4–6.9)
Sex workers	0.7 (4)	0.1 (0.0–0.3)
Other	5.7 (34)	5.5 (3.2–8.2)
**Sexual orientation**		
Homosexual	43.9 (264)	40.2 (34.7–46.4)
Bisexual	54.4 (327)	57.6 (51.4–62.8)
Heterosexual	1.4 (8)	1.8 (0.5–3.9)
Don’t know	0.3 (2)	0.4 (0.01.5)

The majority of MSM believe that vaginal sex is riskier than anal sex, that the risk of HIV is the same with men as with women, and that insertive and receptive anal sex carry the same risks (Table 2). Unprotected sex in the last 12 months was common (65.2%) and among these individuals 90.1% had unprotected sex without a water-based lubricant. Nearly a quarter of MSM engaged in transactional sex in the last 12 months and another 56.2% had sex with a woman. The last female sexual partner for 71.7% of these men was a regular partner and 63.8% did not use a condom at last sex with a woman. HIV testing is low, with 37.4% of MSM never having been tested. Nonetheless, only 17.7% feel they are at high risk of HIV.

**Table 2:** Behavioral & Knowledge Characteristics of MSM in Abidjan, Côte d’Ivoire, 2011–2012 (n = 601).

Variables	Sample Description % (n)	Estimated Population Prevalence % (95% CI)
**Alcohol use**		
Never	27.4 (165)	32.4 (26.0–38.3)
Once a week or less	49.1 (295)	49.1 (43.4–55.6)
More than once a week	23.1 (139)	18.4 (14.5–22.9)
**Non-injection drug use (n = 600)**	9.3 (56)	9.6 (6.2–13.5)
**Consistent condom use during anal sex with men in the past 12 months (n = 585)**	32.6 (191)	34.8 (29.3–41.0)
**Consistent condom and water-based lubricant use with men in the last 12 months (n = 585)**	10.3 (60)	9.9 (6.9–13.7)
**Condom breakage in the last 12 months among those who used condoms (n = 539)**	47.7 (257)	41.7 (35.7–47.3)
**Number of male anal sex partners in the last 12 months (n = 600)**		
(median 4, range 0–96)		
0	2.7 (16)	3.0 (1.2–4.5)
1	22.0 (132)	26.8 (22.0–32.4)
2–3	31.8 (191)	36.5 (31.0–41.9)
4–5	16.7 (100)	15.0 (11.8–19.2)
6–9	11.2 (67)	9.4 (6.1–13.1)
10+	15.7 (94)	9.2 (6.5–12.0)
**Condom use at last anal sex with a man (n = 548)**	70.1 (384)	69.6 (67.7–75.0)
**Sexual position with last two male anal sex partners (n = 585)**		
Insertive	36.9 (216)	41.5 (35.7–48.8)
Receptive	42.1 (246)	40.2 (33.4–46.4)
Both	21.0 (123)	18.3 (13.7–22.7)
**Had vaginal or anal sex with a woman in past 12 months (n = 601)**	50.6 (304)	56.2 (49.8–62.5)
**Type of female partner for last vaginal or anal sex in the last 12 months (n = 304)**		
Regular	66.8 (203)	71.7 (63.3–81.2)
Occasional	30.9 (94)	26.7 (17.1–34.5)
Commercial- I paid her	0.7 (2)	0.6 (0.0–1.8)
Commercial- She paid me	1.6 (5)	0.9 (0.0–3.4)
**Condom use at last sex with a female**	62.7 (190)	63.8 (50.7–74.6)
**Have bought sex in exchange for money or goods in the past 12 months (n = 601)**	11.0 (66)	8.3 (5.5–10.7)
**Have sold sex in exchange for money or goods in the past 12 months (n = 601)**	29.3 (176)	23.4 (19.5–27.2)
**Feel they must hide sex with men when seeking healthcare**	53.6 (322)	55.0 (49.4–60.0)
**History of abuse or harassment because of being MSM**	44.9 (270)	38.5 (32.4–43.6)
**History of forced sex**	24.5 (147)	21.4 (16.6–26.0)
**Knows that having only one sex partner who is HIV- can protect against HIV**	82.5 (496)	82.1 (77.3–86.6)
**Identifies the riskiest sexual partner as:**		
Men	14.5 (87)	12.8 (9.3–16.1)
Women	18.6 (112)	22.9 (17.7–27.9)
Men and women have the same risk	66.1 (397)	63.3 (58.0–69.1)
**Knows that anal sex is the riskiest sexual mode of HIV transmission**	43.4 (260)	38.8 (32.6–44.0)
**Identifies the riskiest type of anal sex to be:**		
Insertive (active) anal sex	5.7 (34)	6.9 (3.9–10.1)
Receptive (passive) anal sex	37.9 (228)	38.5 (32.8–44.2)
Both active and passive anal sex have the same risk	55.6 (334)	53.3 (48.0–59.1)
Both active and passive anal sex have no risk	0.5 (3)	0.6 (0.0–1.4)
**Participated in an HIV awareness session in the past 12 months**	48.1 (289)	39.9 (34.4–45.5)
**Knows an HIV-infected MSM**	20.0 (120)	9.9 (7.2–13.0)
**Self-perceived HIV risk**		
No risk	18.3 (110)	19.6 (15.2–25.3)
Low risk	58.1 (349)	59.1 (53.0–64.5)
High risk	19.5 (117)	17.7 (13.4–22.3)
Already HIV+	3.1 (19)	2.4 (0.9–4.4)
Don’t know	1.0 (6)	1.1 (0.1–2.6)
**Self-reported STD in the last 12 months**	19.0 (114)	19.2 (14.5–24.0)
**Never tested for HIV**	33.8 (203)	37.4 (31.8–43.5)
**Received result of last test (n = 398)**	92.5 (368)	93.9 (90.9–96.6)
**Shared previous HIV test result with sexual partner (n = 368)**	39.4 (145)	40.2 (32.6–48.9)
**Positive HIV test result at study (n = 581)**	19.5 (113)	18.0 (13.0–23.1)
**HIV+ MSM unaware of their serostatus (n = 113)**	84.1 (95)	86.4 (75.1–94.9)

HIV prevalence was 18.0% (95% CI: 13.0–23.1) and 86.4% (95% CI: 75.1–94.9) of infections were newly diagnosed (Table 2). Such infections were defined as an HIV-positive result during the survey among participants who had never been tested or who indicated that their last HIV test result was negative or indeterminate.

### Risk Factors for HIV

In multivariable analysis (Table 3), MSM 25 years or older had 2.62 times the adjusted odds of younger MSM of being HIV infected (95% CI: 1.05–6.57). The odds of being HIV infected decreased as education increased. Alcohol use was not significantly associated with HIV infection (Table 4). No participants reported injection drug use.

**Table 3:** Key Correlates of HIV Infection Among MSM in Abidjan, Côte d’Ivoire, 2011–2012 (n = 581).

Characteristic	Sample HIV Prevalence % (n)	Estimated Population HIV Prevalence (%)	Unweighted OR	Weighted OR (95% CI)	P-Value	Adjusted Weighted OR (95% CI)	P-Value
**Age**							
18–24 years	14.2 (49)	12.5	1	1	0.0157	1	0.0399
25+ years	27.0 (64)	25.8	2.23 (1.18–4.21)	2.44 (1.18–5.02)		2.62 (1.05–6.57)	
**Education level started**							
Never attended	40.0 (14)	38.1	1	1	0.0001	1	0.0388
Primary	30.0 (12)	29.3	0.64 (0.31–1.36)	.68 (0.30–1.54)		0.64 (0.30–1.37)	
Secondary	17.3 (56)	16.1	0.31 (0.17–0.57)	.31 (0.14–0.72)		0.33 (0.13–0.84)	
Post-secondary	17.0 (31)	12.6	0.31 (0.19–0.51)	.23 (0.09–0.63)		0.21 (0.07–0.69)	
**Monthly income in FCFA (500 FCFA = 1 USD)**							
< 25,000	14.5 (33)	13.5	1	1	0.0828	1	0.0223
25,000–49,999	22.7 (34)	28.5	1.73 (1.26–2.38)	2.55 (1.01–6.44)		2.52 (1.03–6.21)	
50,000–149,999	23.2 (32)	16	1.78 (1.30–2.45)	1.22 (0.62–2.41)		1.04 (0.50–2.19)	
150,000+	22.6 (14)	14.6	1.72 (0.64–4.65)	1.10 (0.43–2.78)		0.89 (0.32–2.44)	
**Sexual identity**							
Heterosexual	12.5 (1)	4.2	1	1	<.0001	1	<.0001
Bisexual	16.1 (51)	12.3	1.35 (0.13–13.99)	3.18 (0.43–23.42)		4.04 (0.32–50.27)	
Homosexual	23.9 (61)	25.7	2.20 (0.24–20.47)	7.85 (1.78–34.67)		9.86 (1.19–81.67)	

**Table 4:** Behavioral and Knowledge Correlates of HIV Infection Among MSM in Abidjan, Côte d’Ivoire, 2011–2012 (n = 581).

Characteristic	Sample HIV Prevalence % (n)	Estimated Population HIV Prevalence (%)	Unweighted OR (95% CI)	Weighted OR (95% CI)	P-Value	Adjusted OR (95% CI)	P-Value
**Alcohol use**							
Never	20.0 (32)	17.1	1	1	0.358	1	0.8103
Once a week or less	18.2 (52)	16.3	0.89 (0.62–1.28)	0.94 (0.39–2.29)		1.04 (0.50–2.17)	
More than once a week	21.8 (29)	22.3	1.12 (0.82–1.52)	1.40 (0.67–2.89)		1.25 (0.63–2.49)	
**Have a profile on an MSM website**							
Yes	14.9 (51)	12.5	1	1	0.0001	1	0.0633
No	26.1 (62)	23.3	2.02 (1.53–2.65)	2.12 (1.44–3.12)		1.42 (0.98–2.06)	
**Had vaginal or anal sex with a woman in the past 12 months**							
Yes, with condom at last sex	14.7 (27)	10.1	1	1	0.001	1	0.013
Yes, no condom at last sex	16.5 (18)	19.7	1.15 (0.55–2.39)	2.19 (1.11–4.30)		1.92 (0.97–3.81)	
No	23.6 (68)	21.9	1.80 (1.06–3.06)	2.49 (1.54–4.03)		2.64 (1.38–5.05)	
**Number male anal sex partners in last 12 months (n = 565)**							
1	14.0 (18)	7	1	1	<.0001	1	<.0001
2	13.9 (14)	16.1	0.99 (0.29–3.37)	2.55 (0.62–10.57)		2.18 (0.55–8.73)	
3+	24.2 (81)	25	1.97 (0.94–4.10)	4.44 (2.57–7.68)		4.04 (2.59–6.32)	
**Sexual position with last two male anal sex partners (n = 566)**							
Insertive	8.1 (17)	6.8	1	1	<.0001	1	<.0001
Receptive	26.5 (63)	26.3	4.07 (3.03–5.45)	4.86 (3.37–7.02)		6.36 (3.88–10.44)	
Both	27.7 (33)	24.7	4.33 (2.90–6.47)	4.46 (2.97–6.71)		4.71 (2.69–8.24)	
**Condom use last 12 months during anal sex with men (n = 566)**							
Always	17.4 (32)	11	1	1	0.003	1	0.0047
Not always	21.2 (81)	21.9	1.28 (0.95–1.73)	2.26 (1.32–3.88)		2.31 (1.29–4.12)	
**Condom use at last anal sex with a man**							
Yes	20.1 (74)	17.9	1	1	0.4362	1	0.4312
No	20.5 (33)	21.6	1.03 (0.69–1.54)	1.26 (0.70–2.27)		1.24 (0.73–2.11)	
**Have paid for sex in the past 12 months (n = 565)**							
Yes	26.1 (12)	34.9	1	1	0.0909	1	0.0547
No	19.5 (101)	17	0.68 (0.39–1.20)	0.38 (0.13–1.17)		0.41 (0.16–1.02)	
**Have received something in exchange for sex**							
Never	17.7 (63)	17.1	1	1	0.808	1	0.7207
Yes, not in last 12 months	19.3 (11)	17	1.11 (0.72–1.73)	0.99 (0.46–2.15)		0.71 (0.31–1.64)	
Yes, in last 12 months	23.2 (39)	19.4	1.41 (1.01–1.96)	1.17 (0.60–2.25)		1.07 (0.71–1.610	
**History of physical abuse due to MSM status**							
No	17.0 (85)	15.4	1	1	0.0042	1	0.0061
Yes	35.0 (28)	42.4	2.64 (2.17–3.20)	4.05 (1.56–10.56)		3.66 (1.45–9.23)	
**History of forced sex**							
No	15.6 (69)	14.2	1	1	0.0017	1	0.0058
Yes	31.4 (44)	30.4	2.47 (1.86–3.28)	2.64 (1.44–4.85)		2.54 (1.31–4.93)	
**Ever tested for HIV**							
Yes, received last result	20.0 (71)	17.3	1	1	<.0001	1	0.0003
Yes, did not receive last result	44.8 (13)	53.8	3.25 (1.84–5.75)	5.57 (2.85–10.87)		4.96 (1.86–13.24)	
No	14.7 (29)	14.9	0.69 (0.40–1.20)	0.84 (0.52–1.34)		0.86 (0.53–1.40)	
**Self-reported STD symptoms in last 12 months (n = 580)**							
Yes	26.9 (29)	25.8	1	1	0.2814	1	0.2396
No	17.8 (84)	15.8	0.59 (0.39–0.89)	0.54 (0.18–1.66)		0.52 (0.17–1.55)	
**Self-perceived risk (n = 556)**							
No risk	7.4 (8)	4.2	1	1	0.0002	1	0.0009
Risk	18.5 (83)	18.2	2.84 (1.59–5.07)	5.07 (2.17–11.87)		5.46 (2.01–14.87)	
**Believes HIV messages are relevant to MSM (n = 580**)							
Yes	19.4 (96)	15.8	1	1	0.1526	1	0.0816
No	20.0 (17)	27.5	1.04 (0.59–1.84)	2.03 (0.77–5.35)		2.36 (0.90–6.19)	
**Participated in an HIV education session in last 12 months**							
Yes	20.9 (58)	21.7	1	1	0.1505	1	0.0895
No	18.2 (55)	15	0.84 (0.60–1.17)	0.64 (0.35–1.18)		0.64 (0.38–1.07)	
**Knows anyone with HIV (n = 580)**							
Yes	24.4 (48)	19.4	1	1	0.6543	1	0.5622
No	17.0 (65)	17.1	0.63 (0.46–0.88)	0.86 (0.44–1.69)		0.78 (0.33–1.82)	
**Knows an HIV+ MSM (n = 580)**							
No	16.6 (77)	16.4	1	1	0.0017	1	0.0016
Yes	30.8 (36)	28.7	2.23 (1.71–2.91)	2.06 (1.31–3.24)		2.36 (1.38–4.02)	
**Identifies riskiest sex act as:**							
(n = 568)							
Anal	19.7 (50)	16.2	1	1	0.0039	1	0.0093
Vaginal	14.8 (39)	14.5	0.71 (0.52–0.96)	0.88 (0.57–1.36)		0.75 (0.39–1.41)	
Oral	42.0 (21)	47.2	2.95 (1.98–4.42)	4.63 (1.86–11.54)		4.49 (1.31–15.35)	

As shown in Table 4, not using a condom at last sex was associated with HIV when the sex act was with a woman (AOR 2.64, 95% CI: 1.38–5.05) but not with a man (AOR 1.24, 95% CI 0.73–2.11). Inconsistent condom use with men in the last 12 months increased the adjusted odds of HIV (AOR 2.31, 95% CI 1.29–4.12) and HIV-positive men had more male sex partners than HIV-negative men. HIV was also associated with being the receptive partner (AOR: 6.36, 95% CI: 3.88–10.44) or both the insertive and receptive partner (AOR: 4.71, 95% CI: 2.69–8.24) with the last two sex partners.

The adjusted odds of HIV among MSM who identified oral sex as the riskiest sexual act was 4.49 (95% CI: 1.31–15.35) times as high as for those identifying anal sex as the riskiest act. Self-perceived risk of HIV (AOR: 5.46, 95% CI: 2.01–14.87) and not receiving the result of the most recent HIV test (AOR: 4.96, 95% CI: 1.86–13.24) were both associated with HIV infection.

MSM who had been forced to have sex were more likely to be HIV infected (AOR 2.54, 95% CI 1.31–4.93) as were those who had suffered physical abuse because they are MSM (AOR: 3.66, 95% CI: 1.45–9.23).

## Discussion

The first large-scale bio-behavioral survey of MSM in Côte d’Ivoire, our survey found an HIV prevalence of 18.0% (95% CI: 13.0–23.1) among MSM in Abidjan. This stands in stark contrast to prevalence among general population men in Abidjan (4.1%) and underscores the high risk for and vulnerability to HIV of MSM in Abidjan. (12) The existence of a disparity in the HIV prevalence ratio is consistent with findings from elsewhere; however, the disparity in Abidjan is among the largest in sub-Saharan Africa [20].

A review of the literature suggests that this survey also has the largest single-city sample size of any RDS survey among MSM in Africa. Successful recruitment stems from the value placed on the formative assessment, extensive efforts to engage MSM in the survey planning and implementation process, as well as the lack of laws in Côte d’Ivoire criminalizing homosexual acts.

Reported alcohol consumption and drug use were relatively low compared to other countries [10, 11, 21]. A larger share of MSM than general population men in Abidjan had 2 or more sex partners in the last 12 months (78.0% versus 31.7%) and used a condom at last sex (69.6% versus 46.8%) [22]. Furthermore, the high prevalence of transactional sex in the past year highlights an additional vulnerability to HIV and STIs among MSM in Abidjan.

Our findings that the odds of HIV infection are similar for MSM engaging in exclusively receptive anal sex as for those engaging in insertive and receptive anal sex support similar findings in a review by Beyrer et al [20]. Having more than two anal sex partners in the last 12 months was associated with HIV infection in our survey. While condom use at last sex was not associated with HIV infection, condom use in the last 12 months was, underscoring the importance of asking about both.

The high prevalence of unprotected receptive anal sex without a water-based lubricant further contributes to the high HIV prevalence among MSM. Filling the void of MSM-friendly health services and increasing accessibility of condoms and condom-compatible lubricants could have a substantial impact in reducing HIV transmission among MSM. Condoms and lubricants should be proactively distributed at no cost. Programs targeting MSM should strive for services that are easily accessible, and relevant and acceptable to the client (e.g., non-judgmental, ano-genital STI screening). As access to prevention services is also limited and the majority of MSM feel they must hide their MSM status when seeking healthcare, peers from diverse backgrounds should be engaged to provide prevention services to particularly hidden men. Programs should also include risk reduction concerning condom negotiation skills and sexual position.

The importance of increasing access to regular HIV testing and receipt of test results cannot be underestimated. Though the majority of MSM had been tested for HIV at least once, 86.4% of HIV-infected MSM were unaware of their status, highlighting increased risk for further transmission. Furthermore, the odds of HIV were more than five times as high for MSM who did not collect their last HIV test result as for those who did.

Factors traditionally associated with HIV infection such as older age and inconsistent condom use with men were statistically significant in our study. The high HIV prevalence among MSM < 25 years is extremely concerning and may represent newer infections. The higher HIV prevalence among MSM ≥ 25 years is to be expected given that HIV is a chronic infection.

While general HIV awareness was relatively high among MSM and similar to that found among general population men in the 2011–12 DHS, knowledge of MSM-relevant HIV risk factors was low [22]. Prevalence of such risk factors was concomitantly high. Our findings suggest that MSM engaging in risky sexual behavior may not know their HIV risk or why they are at risk. MSM may not be receiving HIV awareness messages tailored to them, leaving them ill-equipped to make safer sex decisions.

MSM in Abidjan have diverse sexual networks composed of men and women, and commercial and non-commercial partnerships. The share of MSM who had sex with a woman in the last 12 months was similar to findings from Botswana, Namibia, and Malawi [23]. It is possible that many of these men have a female sexual partner to hide their MSM status and cannot use a condom with her lest they seem unfaithful. These men may be especially hidden and less likely to access MSM-specific services. Bisexual activity may form a link between the HIV epidemic in the general and MSM populations, putting both at greater risk. Viral genotype data from other settings suggest that HIV transmission between MSM is not behaviorally segregated from transmission occurring among the general population [20, 24, 25].

Having a profile on an MSM website was negatively associated with HIV infection, possibly because people with online profiles may have more access to online HIV prevention information. HIV status is not provided on most websites used by MSM in Côte d’Ivoire, suggesting that the profiles themselves do not contribute to sero-sorting. Other factors associated with HIV infection include history of forced sex, history of physical abuse due to MSM status, and knowing an HIV-positive MSM. Discrimination and violence related to MSM status have been associated with HIV and shown to hinder service provision [6, 10]. These largely MSM-specific risk factors reveal the additional HIV risks of MSM beyond the biological and require evidence-based structural interventions.

Like many MSM surveys, this study is limited by the challenges of recruiting MSM above age 30 and wealthier MSM [8, 11, 26, 27]. Formative research revealed that these older men exist and are more likely to be hidden and married to women than younger MSM. The greater participation of younger MSM may have biased findings. As older MSM are more likely to be infected with HIV, the true HIV prevalence among MSM in Abidjan may be greater than our estimate of 18.0%. Formative findings indicating additional difficulties in recruiting non-African MSM proved to be true. As interviews were conducted face-to-face, it is possible that there was some underreporting of risk behaviors and awareness of HIV status due to social desirability. Results interpretation is also limited due to the cross-sectional study design. Finally, instructing participants to give coupons to the first three MSM they encounter may have either added randomness to the recruitment process or biased it toward candidates the recruiter sees more often than others.

The high HIV burden among MSM in Abidjan, Côte d’Ivoire is alarming, particularly when compared to men in the general population. In addition to biologic vulnerability, MSM face structural risk factors that require interventions beyond the individual level. While the country has received over $750 million from the United States President’s Emergency Plan for AIDS Relief between 2004 and 2012 and $114 million for HIV from the Global Fund to Fight AIDS, Tuberculosis, and Malaria, access to HIV services among MSM is severely limited and risk behaviors remain high. Côte d’Ivoire’s National Strategic Plan for HIV/AIDS 2011–2015 identifies the need to involve MSM in the HIV response; this study provides an evidence base to strengthen the response. Our experience further indicates that MSM are willing partners in their own health and want to be engaged in efforts to advance HIV prevention, care, and treatment. Programmers and policymakers should harness this energy and involve key MSM stakeholders to inform appropriateness of programming and to discuss mitigating structural determinants of risk. In Abidjan, this may mean increasing access to water-based lubricants, MSM-relevant HIV prevention information, and HIV and psycho-social services for MSM. Our findings also add to the mounting evidence that MSM are at high risk for HIV infection even in generalized epidemics, necessitating a focus on MSM in countries with low and high general population prevalence alike.
